# A Review of Avian Influenza Virus Exposure Patterns and Risks Among Occupational Populations

**DOI:** 10.3390/vetsci12080704

**Published:** 2025-07-28

**Authors:** Huimin Li, Ruiqi Ren, Wenqing Bai, Zhaohe Li, Jiayi Zhang, Yao Liu, Rui Sun, Fei Wang, Dan Li, Chao Li, Guoqing Shi, Lei Zhou

**Affiliations:** 1 School of Public Health, Nanjing Medical University, Nanjing 211166, China; 17807372838@163.com (H.L.); feiwang0331@163.com (F.W.); 2 Public Health Emergency Center, Chinese Center for Disease Control and Prevention, No. 155 Changbai Road, Changping District, Beijing 102206, China; renrq@chinacdc.cn (R.R.); baiwq@chinacdc.cn (W.B.); m381425926@163.com (Z.L.); zjy1950431161@163.com (J.Z.); llly1087@163.com (Y.L.); rui_010527@163.com (R.S.); lidan1@chinacdc.cn (D.L.); lichao1@chinacdc.cn (C.L.); 3 National Key Laboratory of Intelligent Tracking and Forecasting for Infectious Diseases, Chinese Center for Disease Control and Prevention, No. 155 Changbai Road, Changping District, Beijing 102206, China

**Keywords:** avian influenza virus, occupational exposure, exposure patterns, risk factors, prevention and control strategies

## Abstract

Avian influenza viruses (AIVs) pose significant risks to occupational populations engaged in poultry farming, livestock handling, and live poultry market operations due to frequent exposure to infected animals and contaminated environments. This review synthesizes evidence on AIV exposure patterns and risk factors through a comprehensive analysis of viral characteristics, host dynamics, environmental influences, and human behaviors. The main routes of transmission include direct animal contact, respiratory contact during slaughter/milking, and environmental contamination (aerosols, raw milk, shared equipment). Risks increase as the virus adapts between species, survives longer in cold/wet conditions, and spreads through wild bird migration (long-distance transmission) and live bird trade (local transmission). Recommended control measures include integrated animal–human–environment surveillance, stringent biosecurity measures, vaccination, and education. These findings underscore the urgent need for global ‘One Health’ collaboration to assess risk and implement preventive measures against potentially pandemic strains of influenza A viruses, especially in light of undetected mild/asymptomatic cases and incomplete knowledge of viral evolution.

## Simple Summary

Avian influenza virus (AIV) remains a significant threat to public health. Occupational groups with frequent exposure to poultry, livestock, or contaminated environments face a higher risk of AIV infection. This review outlines the key exposure pathways and risk factors for AIV in these populations, including viral characteristics, animal host factors, environmental conditions, and host susceptibility. By synthesizing current evidence, we aim to support the development of targeted prevention strategies to mitigate infection risks and protect occupational workers’ health.

## 1. Introduction

Alpha influenza virus (Influenza A Virus, IAV) is a single-stranded RNA virus with a segmented genome, primarily hosted by wild waterfowl [1]. Based on antigenic differences in surface glycoproteins hemagglutinin (HA) and neuraminidase (NA), IAV can be classified into 18 HA subtypes and 11 NA subtypes. Except for H17N10 and H18N11, which are found only in bats, all other subtypes were initially isolated from avian hosts and are collectively referred to as avian influenza viruses (AIVs) [2].

AIVs can be categorized into highly pathogenic avian influenza viruses (HPAIVs) and low pathogenic avian influenza viruses (LPAIVs). Among them, H5N1 and H7N9 HPAIV pose particularly severe threats to mammals (including humans). According to World Health Organization statistics, from 1 January 2003 to 27 May 2025, 25 countries reported a total of 976 human cases of H5N1 infection, with a fatality rate as high as 48.2% (470/976) [3].

In 1996, Chinese scientists first isolated and identified the highly pathogenic avian influenza H5N1 strain (A/goose/Guangdong/1/1996, Gs/Gd) from geese in Guangdong Province [4]. This strain caused the world’s first human infection cases in Hong Kong in 1997 [5], providing the first confirmation of the virus’s cross-species transmission capability to humans, with the outbreak resulting in 18 human infections and 6 deaths [6]. In 2003, H5N1 outbreaks re-emerged among poultry in China and subsequently spread rapidly to Southeast Asia, Europe, Africa, and Russia. Research demonstrated that domestic duck populations in southern China served as asymptomatic carriers playing a central role in viral transmission, while poultry trade and migratory bird movements were identified as the primary drivers of the virus’s spread across Southeast Asia and globally [7,8,9]. During this period, the virus continued to evolve through genetic reassortment, generating multiple novel genotypes.

The H5N1 clade 2.3.4.4b that emerged in 2020 has continued its global expansion through migratory birds, now reaching Europe, Africa, the Americas, and Antarctica [10]. In late 2021, the Eurasian lineage HPAI H5N1 clade 2.3.4.4b entered North America via wild birds, causing the deaths of millions of poultry and thousands of wild birds in the United States, along with spillover infections in various mammalian species. As of July 2025, over 1000 dairy cattle herds across 17 U.S. states have been infected [11,12]. Recent studies revealed that the North American-reassorted H5N1 virus was introduced into South America in late 2022 and spread among mammals, triggering mass mortality events in sea lions in Peru, Argentina, and Chile [13,14], with estimated deaths of at least 667,000 wild birds and 52,000 wild mammals [15]. More notably, the virus subsequently spread from Argentina to Antarctica, where HPAI H5N1 clade 2.3.4.4b was detected in multiple bird species and two seal species on two Antarctic islands in October 2023 [16]. These epidemiological developments have raised significant concerns among occupational groups including marine biologists, wildlife veterinarians, and zoo/aquarium staff [17].

Epidemiological studies show that before 2024, the main transmission routes of human avian influenza virus infection were direct contact with sick/dead poultry or exposure to live poultry market environments [18,19]. However, in March 2024, the first global case of AIV transmission from dairy cows to humans was reported, significantly broadening the zoonotic transmission pathways of the virus [20]. The latest data indicate that from March to 9 July 2024, the United States has reported a cumulative total of 70 human infection cases related to contact with dairy cows and poultry [6,21], raising high concern in the public health field regarding occupational exposure populations. Occupational exposure groups (such as breeding, slaughtering, and live poultry market workers) face a much higher infection risk than the general population due to prolonged contact with susceptible animals and their excreta [22]. Given the continuous expansion of the host range of H5N1 clade 2.3.4.4b, comprehensive prevention measures must be implemented in wild birds, poultry, and dairy cows to block the spillover transmission of the virus to humans [23].

While existing studies have employed various methodologies—such as market chain analysis, social network analysis, decision trees, scenario modeling, spatiotemporal analysis, and machine learning models [24,25,26,27,28]—to assess risk factors for avian influenza virus emergence and transmission, most remain confined to singular disciplinary approaches. These studies often fail to comprehensively integrate pathogen-, animal-, environment-, and human-related indicators. For instance, some investigations focus solely on viral evolutionary patterns [29], while others restrict their scope to avian influenza exposure in farm settings [30], neglecting to establish connections between these factors and human behavioral risks. Notably, current epidemiological surveys frequently provide only generalized descriptions of case exposures, lacking detailed classification of contact modalities. Compounded by surveillance capacity disparities—particularly lower detection rates in resource-limited regions—reported case data inadequately reflect true infection burdens.

**Figure 1:**
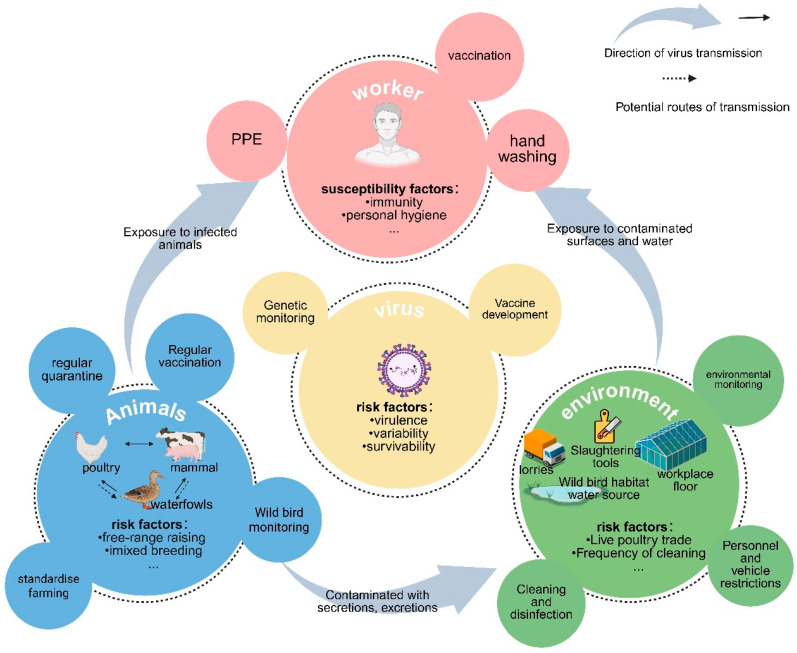
Conceptual framework of AIV transmission risks in occupational populations, integrating viral, host, environmental, and behavioral factors. This schematic illustrates the transmission chain of avian influenza virus (AIV) exposure among occupational populations, comprising three critical components: a. primary exposure routes (direct contact with infected animals or contaminated environments); b. risk determinants (animal husbandry practices and viral characteristics); c. preventive countermeasures (personal protective equipment, immunization, and biosafety management). The diagram employs arrows to delineate transmission pathways, with four central circular zones representing distinct risk dimensions, while subsidiary circles connected by dashed lines correspond to specific intervention strategies (original schematic created with BioRender.com URL (accessed on 11 May 2025).

Based on the theoretical framework of epidemiological triangulation model and by integrating evidence from multidisciplinary studies, this study systematically investigated the exposure pathways and risk factors of avian influenza virus in occupational populations, and deeply analyzed the complex mechanisms of human–animal–environment interactions (Figure 1). The results of this study can provide a scientific basis for the development of precise prevention and control strategies and are of great practical guidance value for reducing the risk of infection in high-risk occupational groups.

## 2. Methods

This study searched academic databases including PubMed, Web of Science, CNKI and WHO official reports from 2000 to 2025. The search strategy combined the following subject terms: (1) Exposure-related terms: “poultry”, “poultry workers”, “live poultry markets”, “influenza A virus”, “H5N1”; (2) Risk factor-related terms: “exposure risk”, “infection risk”, etc. The search cutoff date was 13 July 2025 to ensure inclusion of the latest research evidence. Through comprehensive analysis of occupational exposure risk factors and prevention measure effectiveness, it provides evidence-based support for developing scientific prevention strategies.

## 3. AIV Exposure Patterns Among Occupational Populations

### 3.1. Contact and Respiratory Exposure

Occupational exposure to avian influenza virus (AIV) primarily occurs through the following pathways.

Direct contact with infected poultry or their secretions/excreta is one of the main transmission routes. The World Health Organization (WHO) notes that such contact includes daily management during poultry farming and exposure to viral aerosols, blood, or bodily fluids during poultry processing (e.g., slaughtering, scalding, defeathering, cutting, cleaning, and organ handling) [3,31,32].

Indirect contact transmission is also significant, as workers may contract the virus through contaminated environments (e.g., drinking water, cage cleaning, or poultry manure fertilization) or surfaces (e.g., feed, water sources, poultry housing facilities, or transport vehicles) [3,31,32]. Studies show that AIV can remain infectious for extended periods under suitable environmental conditions, with particularly high detection rates in animal organs, poultry processing surfaces, and cleaning wastewater [33,34]. On 29 March 2023, Chile reported its first confirmed human case of H5N1 infection. Epidemiological investigations revealed the presence of diseased or dead marine mammals and wild birds in proximity to the patient’s residence, suggesting potential environmental exposure as the source of infection [35]. This emerging case further underscores the elevated risk occupational groups face within the avian influenza transmission chain.

Respiratory transmission mainly occurs during slaughtering and close contact with poultry, such as inhaling viral aerosols from feathers or feces during defeathering [36]. These aerosols and droplets can travel 50–80 m through the air [19]. Notably, occupational groups engaged in poultry slaughtering or handling flocks with mortality rates exceeding 10% show significantly higher H5 subtype antibody seropositivity [37]. In practice, poultry workers often face multiple exposure risks simultaneously, with those having prolonged daily contact with diverse poultry species being at particularly high risk [38,39].

Recent studies reveal that direct contact with infected dairy cows or raw milk, along with indirect contact with contaminated environmental objects, has emerged as a novel transmission route for highly pathogenic avian influenza H5N1 virus (HPAIV H5N1). Compared to respiratory transmission, bovine-origin HPAI H5N1 demonstrates greater propensity for transmission through mammary glands in dairy cattle [11]. The virus can remain infectious for up to 3 h on milking equipment surfaces contaminated with raw milk [40], indicating that virus-contaminated equipment and generated droplets during milking pose significant risks to workers. This finding also explains the predominance of conjunctivitis and mild respiratory symptoms among patients in the U.S. outbreak, highlighting the importance of ocular exposure.

### 3.2. Foodborne Exposure

Undercooked poultry products, eggs, and raw milk may become infection sources. Epidemiological reports show that viscera from live poultry after slaughter may still carry infectious virus [41]. Notably, all components of eggs (including shells, yolks, and whites) from infected poultry have tested positive for the virus [42], suggesting the possibility of vertical transmission. The bovine-origin H5N1 virus (clade 2.3.4.4b) exhibits particular affinity for bovine mammary tissue. Infectious viral particles have been detected in raw milk samples from multiple outbreak areas in the U.S., with documented cases of infection in mammals such as cats and raccoons through raw milk consumption [43]. Animal experiments further confirm that bovine-origin H5N1 virus can infect mice and macaques via the digestive tract [44]. However, standardized pasteurization effectively inactivates the virus in dairy products, ensuring consumption safety [45].

### 3.3. Potential Human-to-Human Transmission

Although no direct evidence has yet been found to confirm sustained human-to-human transmission of avian influenza viruses, existing epidemiological data and experimental studies have revealed concerning risk signals. Multiple studies indicate that while sustained human transmission has not been confirmed for subtypes such as H5N1, H7N7, and H7N9, several clustered cases have been observed. For instance, 14 probable limited human transmission events of H7N9 were identified between 2013 and 2017 in China [46]; however, the possibility of common exposure sources cannot be entirely ruled out in these cases [47]. More notably, experimental studies using ferret models (whose respiratory physiology closely resembles humans) have demonstrated that HPAI H5N1 can acquire airborne transmission capability among mammals through adaptive mutations, without requiring reassortment in intermediate hosts [48]. Additionally, human-origin H10N3 viruses have shown limited droplet transmission capacity in mammalian models [49]. Collectively, these findings suggest that avian influenza viruses may gradually accumulate adaptive mutations to gain human transmission potential, highlighting the necessity for enhanced continuous surveillance and risk assessment.

## 4. Risk Factors for AIV Exposure in Occupational Populations

Current human cases of avian influenza remain sporadic, with infection risks resulting from the interplay of multiple factors. From viral characteristics to host factors, environmental conditions, and population susceptibility, these elements intertwine to form a risk network for occupational exposure. A thorough understanding of these risk factors is crucial for developing targeted prevention and control measures.

### 4.1. Viral Factors

#### 4.1.1. Cross-Species Transmissibility and Virulence of Avian Influenza Viruses

**Figure 2:**
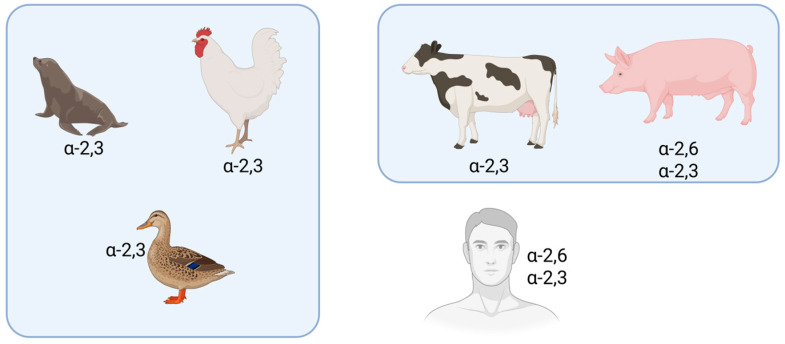
Binding specificity of influenza hemagglutinin (HA) to avian (α-2,3) and human (α-2,6) sialic acid receptors (created with BioRender.com URL (accessed on 16 July 2025).

The cross-species transmission capability of avian influenza viruses primarily depends on their receptor-binding properties. Research shows that avian influenza viruses preferentially bind to α-2,3-sialic acid receptors (avian-type receptors), mainly distributed in avian intestines and the human lower respiratory tract, while human influenza viruses preferentially bind to α-2,6-sialic acid receptors (human-type receptors), predominantly found in the human upper respiratory tract. This difference explains why most U.S. cases of bovine-origin H5N1 infection present with conjunctivitis, as human conjunctival tissue is rich in avian-type receptors [50]. Notably, the respiratory epithelial cells of pigs and certain poultry (e.g., turkeys, quails) express both types of receptors, making them “mixing vessels” for viral gene recombination and significantly increasing the risk of cross-species transmission [51,52] (Figure 2). On 30 October 2024, the U.S. Department of Agriculture confirmed the first case of H5N1 infection in pigs in Crook County, Oregon—a breakthrough discovery with significant public health implications that demands heightened vigilance and sustained monitoring [43].

Although bovine-origin H5N1 currently retains primarily avian-type receptor binding properties, studies show that a single Gln226Leu mutation can confer full human-type receptor binding capability. More concerning, bovine respiratory and mammary epithelial cells express low levels of human-type receptors, providing a potential environment for viral adaptation [53]. These findings highlight the evolutionary risks of H5N1. Indeed, adaptive mutations and genetic recombination are key drivers of avian influenza virus evolution, significantly enhancing their ability to infect mammals, including humans. The 2013–2017 H7N9 outbreak exemplifies this pattern, where multiple rounds of recombination led to markedly increased pathogenicity, resulting in 290 deaths during the fifth wave [54]. Of particular concern is the potential recombination between seasonal human H1N1/H3N2 viruses and the currently circulating H5N1 clade 2.3.4.4b, which could produce novel strains with both high pathogenicity and efficient human-to-human transmission—considered the greatest pandemic risk posed by H5 subtypes.

While most reported human cases are mild, studies show the virus has a unique tropism for feline central nervous systems [43], with neurological symptoms observed in infected goats, otters, dolphins, sea lions, and other mammals [55]. These findings underscore the need for continued vigilance regarding neurotropic variants and their potential public health impacts.

#### 4.1.2. Viral Survival Capability

Waterfowl feces contain high viral loads, with the virus remaining infectious for at least 30 days at 4 °C [56]. Suitable temperatures prolong viral survival—4 days in water at 22 °C versus 30 days at 0 °C [57]. Gene fragments of bovine-origin HPAI H5N1 retain infectivity for 20 days at 4 °C [44]. Modeling shows summer temperatures reduce environmental AIV contamination risks by 70–90% [58]. Additionally, animal experiments confirm that aerosolized influenza virus particles remain more stable at lower relative humidity (20–40%), enhancing transmission and infectivity [59]. The winter–spring peak of human H7N9 infections in warm, humid southern regions further demonstrates the correlation between environmental survival and transmission capacity [60].

### 4.2. Animal Host Factors

#### 4.2.1. Wild Bird Activities

Animal hosts play a pivotal role in viral transmission chains. Wild birds, particularly migratory species, serve as crucial vectors for cross-regional virus dissemination, as infected individuals can shed the virus through saliva, mucus, and fecal excretion [61]. Genetic analyses indicate that long-distance migration of wild birds was the primary driver of HPAIV H5N1 geographic dispersion in China’s Poyang and Qinghai lake regions [4]. Moreover, HPAI H5N1 outbreak risks correlate with proximity to wild bird migration routes and vegetation coverage [62] as wetlands and nature reserves attract higher waterfowl densities [44].

The overlap between urbanization/agriculture and wild bird habitats increases opportunities for avian influenza virus spillover. Most outbreaks can be traced to introductions by wild birds, potentially through multiple events. Studies show open water bodies within 500 m of free-range poultry farms are major introduction pathways [63,64], with farmland ranking second only to wetlands/natural vegetation for outbreak risks [62].

#### 4.2.2. Poultry Trade

The live poultry trade chain (breeding, transport, and sales) plays a significant role in AIV transmission and spread. Backyard production systems increase contact between wild birds, domestic poultry, livestock, and humans, particularly elevating exposure risks for small-scale farmers [65,66]. Shared water sources with wild ducks—which have low vaccination rates (<30%) and carry diverse AIV subtypes similar to wild waterfowl [66]—facilitate fecal–oral transmission [67,68], prolonged viral shedding [69,70], and cryptic circulation in mixed poultry populations. Vietnamese and Korean studies both associate HPAI outbreaks with high free-grazing duck densities, while Korean research identifies duck farm density and proximity to live poultry markets as major risk factors [71].

Live bird markets (LBMs) represent critical human–animal interfaces and potential hubs for AIV accumulation, amplification, and sustained transmission. Viral detection rates increase tenfold from farm to retail settings [72]. Transport durations exceeding three hours elevate LBM outbreak risks, as stressed poultry become more susceptible [73]. LBMs mix large numbers of untested birds from multiple sources, creating recombination hotspots [74]. Bangladeshi research found slaughter areas three times more contaminated than holding areas [58], with unpartitioned birds exposed to high viral loads from slaughter waste and aerosols. High stocking densities (>10 birds/m^2^) and overnight market stays further increase transmission risks [73].

#### 4.2.3. Milking Activities

Research indicates the B3.13 H5N1 genotype first detected in U.S. dairy cows in early 2024 originated from multiple wild bird introductions and spread through milking practices [50]. Unlike other mammals that develop severe symptoms, H5N1 in cows primarily causes subclinical manifestations (e.g., reduced milk yield, mastitis), complicating early detection. The CDC has reported 41 cow-exposure cases [75], with milking, manure handling, and transport posing the highest risks [76]. Dairy workers with direct raw milk contact face particularly elevated infection risks.

### 4.3. Environmental Factors

#### 4.3.1. Mechanical Carrying by Personnel and Production Materials

Workers, visitors, and middlemen serve as important virus introduction pathways. For example, failure to change clothing, carrying personal items (e.g., phones, jewelry), or omitting showers when entering/exiting farms may increase transmission risks [77,78]. Middlemen linking farms, LBMs, and vendors facilitate cross-transmission [79]. Shared vehicles, cages, and egg trays are important AIV transmission vehicles [63], with tires potentially carrying wild bird feces [80]. Transport of infected cows, shared equipment, vehicles, and personnel are likely routes for inter-farm spread [81].

#### 4.3.2. Environmental Contamination Factors

Poultry activities (breeding, slaughter) pollute surrounding environments. Even healthy birds can carry and shed AIV [36]. Cambodian LBMs showed airborne AIV originating from poultry, while Changsha, China, detected AIV in 90.91% of drinking water and 68.49% of cage swab samples [82]. Poultry breeding and slaughter operations amplify contamination ranges. Poor manure management exacerbates farm-to-farm transmission [83]. In China’s Poyang Lake region, farmers’ use of untreated feces as fertilizer or sales to aquaculture (e.g., fish/pearl farming) caused environmental contamination [84]. Inadequate disinfection (e.g., ≤1/week in LBMs) and poor ventilation increase viral accumulation [73,85].

### 4.4. Susceptible Population Factors

Human susceptibility to AIV and infection severity correlate with immune status. Underlying conditions (e.g., obesity, diabetes, cardiovascular disease, chronic lung disease, immunosuppression) increase H7N9 infection risks. Some AIV subtypes show distinct gender differences (e.g., male H7N9 incidence double females), possibly reflecting exposure frequency variations [51,86]. Current bovine H5N1 cases primarily affect U.S. adults exposed to cattle infected with highly pathogenic avian influenza virus [87]. Ferret models demonstrate that prior H1N1 infection can induce cross-reactive antibodies against H5N1 (clade 2.3.4.4b), significantly reducing respiratory viral replication and blocking contact transmission [88].

## 5. Prevention and Control Measures for Occupational AIV Exposure

### 5.1. Multi-Dimensional Surveillance Network and Response

Establishing comprehensive animal disease surveillance systems is critical for interrupting influenza virus transmission chains. Regular health checks, screening, and vaccination of livestock can effectively maintain herd health and enable early infection detection [43]. Studies show poultry immunization coverage exceeding 80% reduces flock outbreak incidence [38]. Routine environmental monitoring for AIV is equally vital for genomic surveillance. The international community urgently needs mechanisms for rapid sharing of clinical samples, viral isolates, and genetic sequences of novel strains. Retrospective analyses suggest the virus may have been circulating in U.S. dairy cows as early as December 2023 [89], highlighting the importance of environmental surveillance. Wastewater monitoring has proven effective for tracking HPAIV spread, with H5-type virus detected at 303 U.S. monitoring sites from 7 to 20 July 2024 [11]. Since low-pathogenicity AIV often circulates cryptically—with clinical signs appearing only after widespread transmission—enhanced active animal and environmental surveillance is decisive for outbreak control.

### 5.2. Enhanced Environmental Management and Hygiene

Scientific farm planning can reduce wild bird contact. Locating farms away from wild bird habitats, implementing single-species rearing, and avoiding mixed livestock/poultry farming minimize cross-species infection risks.

Strengthening personnel/vehicle entry protocols and facility sanitation are key animal health protection measures [43]. Field evidence confirms large-scale farms using footbaths, concrete floors, and frequent cleaning significantly reduce AIV outbreak risks [90]. Notable biosecurity standard disparities exist across livestock sectors: poultry farms typically implement stringent systems (closed management, mandatory disinfection, regular vaccination), while dairy farms’ lower standards (open rearing, lax disinfection, inadequate vaccination) increase avian influenza transmission risks—requiring special attention [90].

LBMs “clean days” and “zero inventory” policies effectively improve hygiene [91,92]. Promoting the “large-scale farming, centralized slaughtering, cold-chain distribution, chilled marketing” model reduces risks. Research shows closing LBMs during H7N9 outbreaks reduced daily infections by 97–99% [93]. Enhanced ventilation reduces airborne pathogen concentrations, with Chen Wei et al. demonstrating that optimized market airflow effectively decreases AIV transmission [94].

### 5.3. Health Education, Occupational Training, and Worker Symptom Monitoring

Disseminating AIV protection knowledge and standardizing personal protective equipment (PPE) use are essential. Meta-analyses confirm that biosecurity measures—particularly handwashing and PPE use—are core AI control strategies [19]. Standard PPE should include fluid-resistant coveralls, N95 masks, goggles, gloves, and boot covers. During the 2024 U.S. H5N1 outbreak, most patients lacked face shields or goggles [11]. Proper hygiene practices depend on accurate AIV knowledge [95]. While smallholders understand the need for masks/handwashing, they rarely use them during poultry handling or coop cleaning [96], underscoring the need for enhanced occupational education.

Promoting avian influenza vaccination is crucial. H7N9 vaccination can prevent viral recombination and reduce human-type receptor binding capability [97]. H5N1 human vaccine development has achieved major breakthroughs, with several candidates now available. Finland, Austria, and other European countries have already vaccinated farm workers against H5N1 to prevent potential pandemics [4].

To enhance the effective surveillance of avian influenza virus infections in humans, it is imperative to strengthen routine symptom monitoring among high-risk occupational groups while improving screening mechanisms for severe respiratory diseases of unknown origin in healthcare institutions. However, current surveillance data may significantly underestimate the actual infection burden, as most infected individuals present with only mild or asymptomatic manifestations, making confirmed cases merely the “tip of the iceberg” of the true infection situation. The influenza-like illness (ILI) surveillance system in sentinel hospitals plays a pivotal role in early detection of such viruses, with existing evidence demonstrating its crucial value in identifying human H9N2 infection cases [98]. Therefore, there is an urgent need to comprehensively enhance the early warning capacity of the surveillance system through measures including expanded monitoring coverage, optimized testing strategies, and improved reporting sensitivity.

## 6. Conclusions and Prospects

Since the first reported human case of H5N1 infection in Hong Kong in 1997, the risk of cross-species transmission of avian influenza viruses has continued to escalate. The recent confirmation of a novel transmission pathway from ruminants to occupational populations has further highlighted the significant health threats faced by exposed workers. This study comprehensively analyzes the primary exposure routes of AIV among occupational groups, demonstrating that infections originate from direct contact with infected poultry, dairy cattle, or contaminated environments, followed by viral entry through mucosal (ocular, nasal, or oral) contact or respiratory routes. Guided by the “One Health” concept, we innovatively developed a four-dimensional occupational exposure risk assessment framework incorporating viral molecular characteristics, host animals, human susceptibility, and environmental factors, confirming that avian influenza epidemics result from multifactorial interactions.

Regarding prevention strategies, this study proposes tiered recommendations. The primary objective is establishing a coordinated surveillance network spanning animal-human–environment interfaces for early outbreak detection, followed by enhanced implementation of biosecurity measures, particularly targeting high-risk exposure scenarios. These findings provide critical scientific foundations for improving avian influenza control systems.

For prevention strategies, we propose tiered recommendations: The primary task is establishing coordinated animal–human–environment surveillance networks for early outbreak detection, followed by focused enhancement of biosecurity implementation, particularly for high-risk exposure scenarios. These findings provide important scientific foundations for improving avian influenza control systems.

It must be emphasized that occupational prevention strategies must balance scientific rigor with practical feasibility while accounting for regional disparities. Resource allocation should prioritize intelligent monitoring technologies and integrated supply chain management in high-income regions, optimized resource distribution to protect core farming areas and live poultry markets in middle-income areas, and basic sanitation infrastructure and occupational protection in low-income settings. Vaccination strategy optimization represents the cornerstone of prevention systems, recommending risk-stratified approaches prioritizing high-risk occupational groups (breeders, slaughterhouse workers, veterinarians) alongside intensified vaccine development and efficacy evaluation.

Future research should focus on fundamental studies elucidating molecular mechanisms and evolutionary patterns of cross-species transmission, applied research developing occupational exposure risk assessment tools and protective technologies for high-risk occupations, and implementation science conducting cost-benefit analyses and intervention optimization across resource settings.

## 7. Limitations

This occupational exposure risk framework, based on systematic literature review, has several limitations. First, current studies inadequately quantify exposure levels, particularly lacking detailed analyses of job-specific exposure characteristics. Second, most data derive from high-income countries, potentially misrepresenting risk profiles in developing nations. Finally, understanding of emerging exposure routes (e.g., bovine-associated transmission) remains preliminary. Future field epidemiological investigations and exposure assessment studies are required to refine occupational risk evaluation systems.
